# Veno-arterial extracorporeal membrane oxygenation as a perioperative support to redo cardiac surgery for inoperable adult patients: a case series

**DOI:** 10.1093/ehjcr/ytad569

**Published:** 2023-11-16

**Authors:** Alvaro Diego Peña, Alejandro Moreno-Angarita, Mayra Estacio, Diego Fernando Bautista, Ivan Fernando Quintero, Stephany Olaya, Eduardo Alberto Cadavid

**Affiliations:** Departamento de Cirugía, Servicio de Cardiovascular, Fundación Valle del Lili, Cali, Colombia; Departamento de Cirugía, Servicio de Cardiovascular, Fundación Valle del Lili, Cali, Colombia; Centro de Investigaciones Clínicas, Fundación Valle del Lili, Cali, Colombia; Departamento de Medicina Interna, Fundación Valle del Lili, Cali, Colombia; Medicina Crítica, Cuidado Intensivo Adultos, Fundación Valle del Lili, Cali, Colombia; Anestesiología, Anestesiología Cardiovascular, Fundación Valle del Lili, Cali, Colombia; Departamento de Cirugía, Servicio de Cardiovascular, Fundación Valle del Lili, Cali, Colombia; Departamento de Cirugía, Servicio de Cardiovascular, Fundación Valle del Lili, Cali, Colombia

**Keywords:** Inoperable, VA-ECMO, Cardiogenic shock, Redo cardiac surgery, Valvular disease, Case series

## Abstract

**Background:**

The present article describes three cases of patients in cardiogenic shock (CS) with previous cardiac surgery that made them initially inoperable. Perioperative support with veno-arterial extracorporeal membrane oxygenation (VA-ECMO) improved haemodynamic status and results in these high-risk patients.

**Case summary:**

Case 1 is a 57-year-old male morbidly obese with previous aortic valve replacement (AVR) who presented with chest pain and developed cardiac arrest. Cardiopulmonary resuscitation and femoral VA-ECMO were initiated. Three days later, a redo AVR was performed. Veno-arterial extracorporeal membrane oxygenation was maintained for 12 days, followed by 7 days of veno-venous ECMO for complete recovery. Case 2 features a 39-year-old male with two previous mitral valve replacements (MVRs). The first is due to papillary muscle rupture, and the second is due to endocarditis of the mitral prosthesis. He presented with CS and pulmonary oedema. Emergency surgery was performed and the patient was then placed in VA-ECMO. Weaning off was achieved 3 days after surgery. Case 3 is a 21-year-old female with a previous MVR due to rheumatic disease. She presented with CS, severe mitral prosthesis stenosis, and a pulmonary embolism. Femoral VA-ECMO was initiated, and one day later, she underwent a redo MVR operation. Extracorporeal membrane oxygenation was discontinued 4 days later.

**Discussion:**

Dysfunctional prosthetic valves leading to CS may benefit from a redo cardiac operation supported by a perioperative VA-ECMO to optimize haemodynamic status. Despite the results from risk prediction scores, this approach has the potential to reduce operative mortality in initial inoperable patients and allow a definitive redo cardiac surgery.

**Learning points**

- Veno-arterial extracorporeal membrane oxygenation serves as a perioperative support strategy to reduce operative mortality in high-risk patients in order to perform a redo cardiac surgery.
- Risk prediction scores should not be used in isolation but rather as a comprehensive assessment of the patient’s status and response to therapy.

## Introduction

In cardiac surgery, an inoperable patient is one who is not a candidate for surgical intervention due to the severity of their condition or other comorbidities that make initial surgery unsafe. This condition has been described in some clinical trials.^1,2^ There is not currently a standard definition for inoperable patients with cardiogenic shock (CS) and bioprosthetic valve dysfunction. In such cases, extracorporeal membrane oxygenation (ECMO) therapy may be considered a means of stabilizing unstable patients and reducing the risks associated with surgical intervention. The utilization of short-term mechanical circulatory support has increased in the last 10 years, leading to reduced mortality rates among severely compromised patients.^3^ Veno-arterial ECMO serves as a bridge for three central indications: a bridge to recovery, a bridge to decision-making, and a bridge to ventricular assist device or heart transplantation. One emerging role is the use of ECMO to stabilize patients with circulatory failure and multi-organ dysfunction before definitive treatment with cardiac surgery.^3^

Veno-arterial ECMO allows for the postponement of definitive surgery by several days or weeks, facilitating the achievement of circulatory stability. However, the literature supporting these indications for ECMO is limited and mainly focuses on first cardiac surgeries not cardiac re-operations. Management decisions are typically made on a case-by-case basis.^3^

In this case series, we present three cases in which VA-ECMO was utilized as a perioperative support to surgery in patients with prior cardiac valve surgery who developed CS and were deemed inoperable. The use of VA-ECMO allowed for the stabilization of these critically ill patients and subsequently facilitated successful cardiac surgical intervention.

## Summary figure

**Table:** 

Events	
**Patient 1**	
Admission: 16 September	CS due to acute myocardial infarction with ST elevation, thrombolysis with alteplase due to valvular thrombosis, and heart arrest.
17 September	VA-ECMO support with peripheral cannulation, lliac injury and repair during cannulation, and heart arrest.
19 September	Redo surgical valvular replacement (SVR) aortic with mechanical valve 21 mm.
28 September	Heart function recovery and switch to veno-venous (VV)-ECMO support.
5 October	De-cannulation of VV-ECMO.
Discharge: 4 November	Hospital discharge with outpatient management.
**Patient 2**	
Admission: 19 March	CS and pulmonary oedema due to mitral valve prosthetic ring dehiscence, IABP support.
20 March	Emergent redo mitral SVR with mechanical valve 31 mm, VA-ECMO support with peripheral cannulation.
23 March	De-cannulation of VA-ECMO.
Discharge: 21 April	Hospital discharge with outpatient management.
**Patient 3**	
Admission: 19 July	CS and pulmonary oedema due to bioprosthetic mitral valve with critical stenosis.
31 July	VA-ECMO support with peripheral cannulation.
2 August	Redo mitral SVR with mechanical valve 25 mm.
3 August	De-cannulation of VA-ECMO.
Discharge: 28 September	Hospital discharge with outpatient management.

## Cases description

### Patient 1

**Figure 1:**
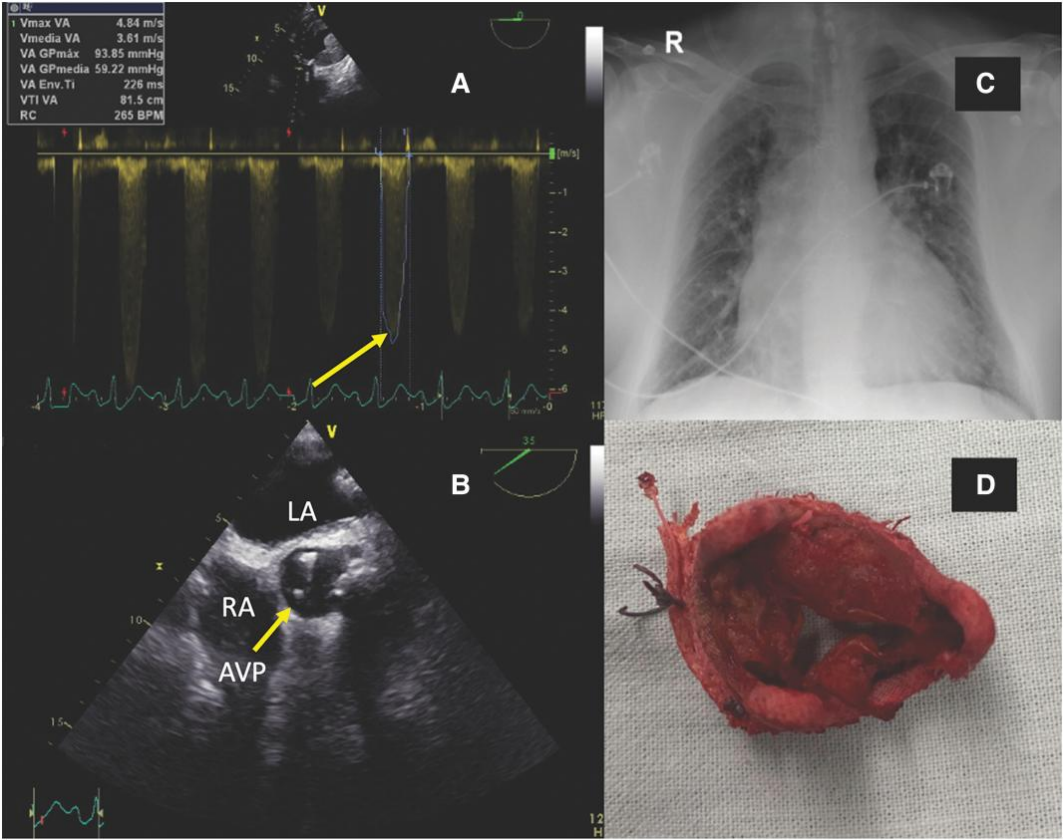
Case 1: (*A*) Continuous spectral Doppler in transoesophageal echocardiogram and deep transgastric projection of the aortic prosthesis, with high velocities and gradients (peak velocity of 4.8 ms, mean gradient of 59 mmHg, and acceleration time of 90 ms). (*B*) Short-axis transoesophageal echocardiogram in mid-oesophagus at the level of the aortic valve prosthesis. (*C*) Bilateral alveolar opacities. (*D*) Dysfunctional bioprosthetic aortic valve causing cardiogenic shock. AVP, aortic valve prosthesis.

Case 1 is a 57-year-old male with a history of hypertension, obesity class III, and aortic valve replacement (AVR). His medications included valsartan, amlodipine, metoprolol, and aspirin. He presented to the emergency department with acute-onset chest pain, dyspnoea, tachypnoea, and rales on examination. The initial electrocardiogram showed heart rate of 100 b.p.m., ST elevation and hyperacute T waves in V1-3, AVR, and reciprocal ST depression in I, II, III, and AVF-electrocardiogram is provided in the supplementary Appendix. Chest radiography showed pulmonary oedema signs. The patient was urgently taken to the cardiac catheterization lab; coronary angiography did not show significant lesions. This was followed by ventricular tachycardia and cardiac arrest. Transthoracic echocardiography showed a calcified bioprosthesis with critical stenosis and thrombosis (*Figure 1A* and *B*). It was decided to perform emergency thrombolysis, which was unsuccessful, and the patient presented CS and multi-organ dysfunction (*Table 2*). Veno-arterial ECMO was used as a bridge to recover from shock. A vascular injury occurred during percutaneous femoral cannulation, requiring external iliac artery exposure and repair. After 3 days of VA-ECMO support and haemodynamic stabilization, a redo AVR (*Figure 1D*) was performed with a prosthetic mechanical valve. The patient’s cardiac function improved after 12 days of VA-ECMO support, but he experienced persistent respiratory failure due to ventilator-associated pneumonia (VAP), requiring VV-ECMO. After a total of 18 days of ECMO support, the patient was successfully de-cannulated and discharged for further outpatient management.

### Patient 2

**Figure 2:**
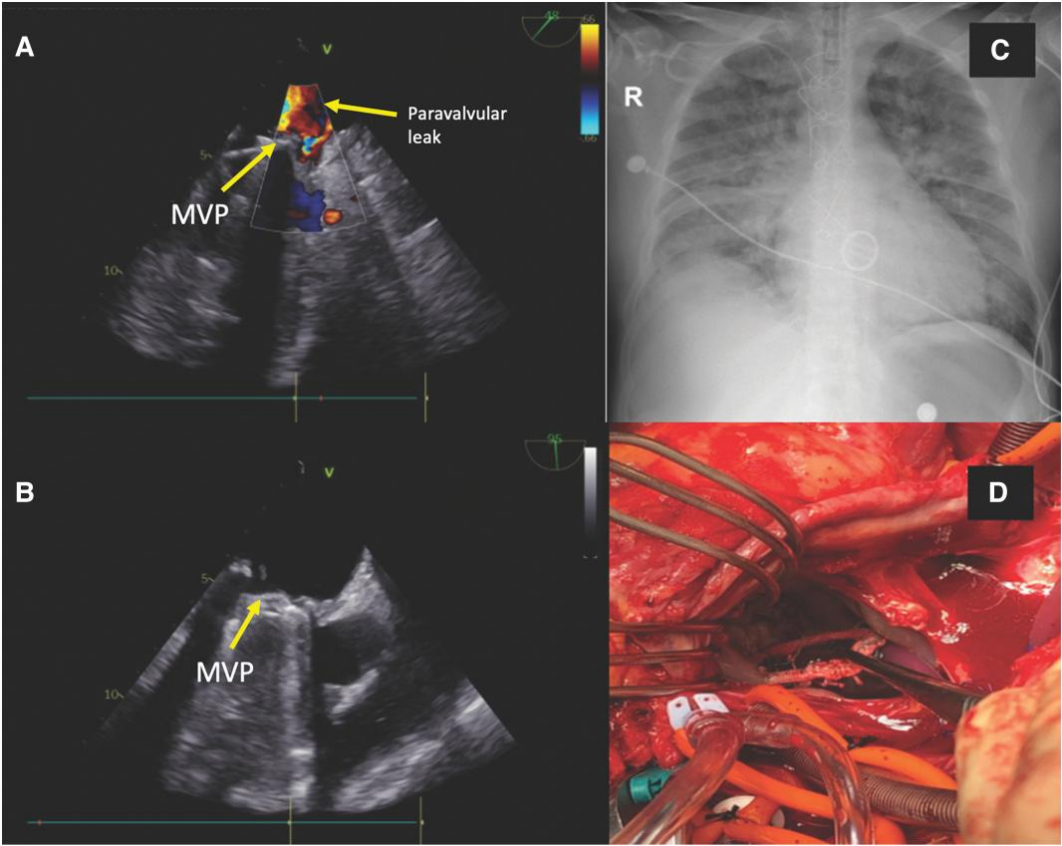
Case 2: (*A* and *B*) Transoesophageal echocardiogram in mid-oesophageal projection at the level of the mitral prosthesis. Doppler (*A*) shows moderate paravalvular leak due to detachment of the posterior mitral ring. (*B*) Two-dimensional model shows opening and closing of the mitral valve discs. (*C*) Increase in size of pulmonary hilum due to pre-capillary pulmonary hypertension, pulmonary oedema with bilateral central predominance opacities, and right pleural effusion or thickening. (*D*) Atrial septal approach for mitral valve replacement: mechanical mitral valve dehiscence due to endocarditis leading to shock.

A 39-year-old man with a history of severe mitral insufficiency due to papillary muscle rupture underwent mechanical mitral valve replacement (MVR), requiring a prolonged intensive care unit (ICU) stay and treatment for bacteraemia. Three months later, he developed bacterial endocarditis of the mitral valve prosthesis and underwent a second MVR with a 31 mm mechanical valve. Ten months later, he presented to the emergency department with orthopnoea, oedema, hypotension, and oliguria (*Figure 2C*). His medications included furosemide, carvedilol, and warfarin. The emergency transoesophageal echocardiogram revealed severe mitral regurgitation due to mitral valve prosthetic ring dehiscence (*Figure 2A* and *B*). Although planned for VA-ECMO support before surgery, the patient’s condition rapidly deteriorated with persistent tissue hypoperfusion (*Table 2*), and the decision was made to use cardiopulmonary bypass via femoral cut-down. A third redo MVR was achieved (*Figure 2D*), and then VA-ECMO was initiated. After 4 days, he was successfully de-cannulated and discharged from the hospital after 11 days. He received an additional 6 weeks of outpatient antibiotic treatment.

### Patient 3

**Figure 3:**
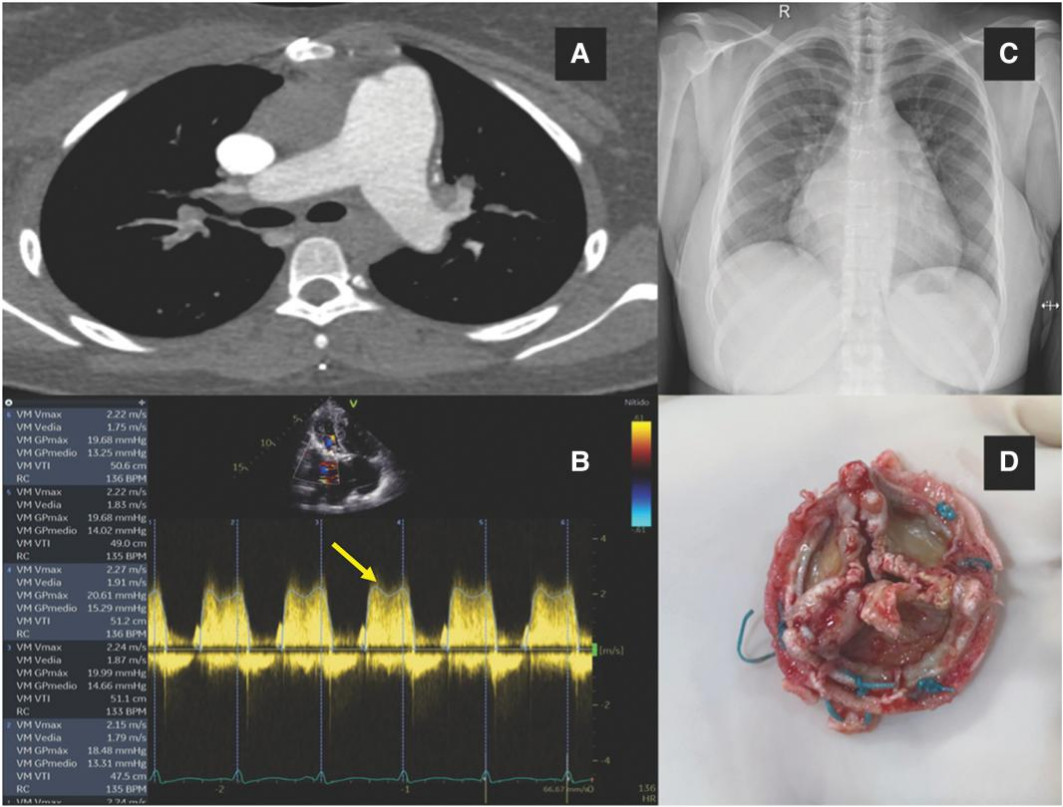
Case 3: (*A*) Pulmonary embolism in the artery for the anterior segment of the left upper lobe and apical region of the right upper lobe. (*B*) Transthoracic echocardiogram in apical four-chamber view. Continuous spectral Doppler of the mitral valve prosthesis (arrow) with high gradients and velocities (maximum velocity of 2.2, mean gradient of 15 mmHg, and mean pressure time of 180 ms). (*C*) Increased cardiac silhouette. Symmetrical pulmonary hila with prominence of vascular markings. (*D*) Explanted mitral valve bioprosthesis showing marked thickening of its leaflets and restriction to opening and closure.

A 21-year-old female patient with a history of rheumatic fever and a bioprosthetic MVR in 2015 presented with dyspnoea, orthopnoea, and fatigue. She regularly took aspirin and her physical examination findings showed signs and symptoms of congestion and hypoperfusion. Transthoracic echocardiography revealed a bioprosthetic mitral valve with critical stenosis (*Figure 2B*). In addition, pulmonary thromboembolism is diagnosed (*Figure 3A*). A decision is made for thrombolysis, with a transient improvement. Cardiogenic shock and multi-organ dysfunction developed 48 h later. The patient required VA-ECMO support as a bridge to surgery. After 3 days of support, a redo MVR was performed with a 25 mm mechanical valve. After de-cannulation, she was complicated by *Klebsiella pneumoniae* septicaemia, which was treated with broad-spectrum antibiotics for 18 days, and the patient was later discharged.

All the information regarding the operative variables are provided in *Table 4*. Follow-up with the patients was conducted via telephone approximately 1 year after the surgery. As of the present date, the first and third patients are living without complications or cardiac-related symptoms. The second patient died in the subsequent year due to new valvular complications.

## Discussion

In selected cases, ECMO can be utilized as a bridge to prepare critically ill patients for high-risk cardiac surgical operations, such as cardiac redo operations. The decision to perform emergent cardiac valve replacement in the context of redo cardiac surgery and CS is complex and multi-factorial since it is associated with extremely high operative mortality.^4^ In the literature, the term inoperable has been used based on a predicted risk of mortality determined by the Society of Thoracic Surgeons (STS) greater than 50% at 30 days. Other factors such as frailty, advanced age, or severe comorbidities also influenced the classification. The ACCF/AATS/SCAI/STS Expert Consensus^5^ clarifies that there is no established definition of ‘inoperable’, but the decision of the best treatment option for a certain risk profile is under the surgeon’s judgment.

In our cases, the initial patient’s evaluation classified them as inoperable because of highly unstable haemodynamic conditions, the use of recent thrombolysis, a high score in the risk prediction scores (*Tables 1* and *2*), and previous cardiac surgery. The mortality prediction after initial surgery was greater than 50% under EuroSCORE 2, and the calculated probability of survival after VA-ECMO support was less than 50% according to the Survival After Veno-Arterial-ECMO (SAVE) score. Prior to surgery, VA-ECMO provided the opportunity to rectify CS and re-evaluate the risk profile in order to pursue a final redo of cardiac surgery. The decision to initiate ECMO support was made by a multi-disciplinary team. In our experience, two out of three cases were placed under percutaneous VA-ECMO prior to surgery, improving their physiological condition according to the SOFA score calculated before ECMO support and before definitive surgery (*Table 2*). In our second case, due to the patient’s rapid deterioration, it was necessary to perform a salvage surgery and then place the patient on post-cardiotomy VA-ECMO support.

**Table 1:** Haemodynamic status before extracorporeal membrane oxygenation support

	Case #1		Case #2	Case #3		Normal ranges
	Before ECMO	Before surgery	Before ECMO	Before ECMO	Before surgery	
Hb (mg/dL)	20.2	10.7	8.3	8.8	9.6	13.7–17.5 mg/dL
Lactate (mmol/L)	3.2	2.1	3.4	4.7	1.6	05–1.6 mmol/L
pH	7.38	7.46	7.48	7.32	7.51	7.35–7.45
PCO2 (mmHg)	34	42	32	23	32	32–48 mmHg
PO2 (mmHg)	79	104	77	75	199	83–108 mmHg
HCO3 (mmol/L)	22.7	29.9	25.5	11.9	25.5	22–26 mmol/L
LVEF (%)	30	30–35	80	65	—	
Creatinine (mg/dL)	1.79	0.78	1.64	1.80	0.37	0.67–1.17 mg/dL
Total bilirrubin (mg/dL)	2.59	2.07	2.65	5.57	18.09	0–1.4 mg/dL
Dialysis (yes/no)	Yes		Yes	Yes		
CPR (yes/no)	Yes		No	No		
AF (yes/no)	No		No	No		

**Table 2:** Risk prediction scores

Patient number	EuroSCORE 2	Veno-arterial-ECMO (SAVE) score		SOFA score
# 1	55.99%	Risk Class IV (−8)	Survival 30%	Pre-ECMO 10 pts Pre-surgery 9 pts
# 2	59.68%	—	—	Post-cardiotomy 9 pts
# 3	59.22%	Risk Class III (−3)	Survival 42%	Pre-ECMO 10 pts Pre-Surgery 2 pts

Regarding the duration of ECMO, data from the ELSO registry indicate that survival rates increase up to 96 h of support, plateauing until Day 12. Afterward, survival decreases significantly due to an increased risk of coagulopathy, infection, and thrombosis.^6^ Despite this, there are no recommendations for a specific duration or weaning strategy to increase survival, as the required treatment duration depends on the underlying pathological process.^6^ In two out of three cases, the duration was within the survival peak of 4 days (*Table 3*). However, in the first case, support was prolonged up to 19 days (12 VA and 7 VV) due to refractory multi-organ failure and the need for bipulmonary support due to sepsis while ensuring no major complications that would prohibit continuation. The duration of VA-ECMO was individualized based on the patient’s response to therapy and overall clinical status. Hybrid ECMO configurations have been described in the Chinese registry,^7^ including conversion from VA- to VV-ECMO. Data analysis shows that dynamic conversion is associated with lower hospital mortality compared with VA-ECMO alone. Our first patient underwent this conversion successfully due to severe respiratory failure from nosocomial pneumonia after cardiac function recovery.

**Table 3:** Perioperative extracorporeal membrane oxygenation support

	Case #1	Case #2	Case #3
Total time on ECMO (days)	12 AV–7 VV	4 AV	4 AV
ECMO pre-operative (days)	2	0	3
ECMO post-operative (days)	17 (10 AV–7 VV)	3 AV	1 AV
Route of cannulation	Peripheral	Peripheral	Peripheral
ICU stay (days)	39	11	47
Cause of CS	Left ventricular failure due to thrombosis of prosthetic aortic valve	Paravalvular mitral leakage due to infectious endocarditis	Right heart failure due to severe mitral stenosis and pulmonary embolism

**Table 4:** Operative variables

	Case #1	Case #2	Case #3
First surgery	Tissue aortic valve replacement	Mechanical mitral valve replacement	Bioprosthetic mitral valve replacement
Last surgery	Redo-SVR Ao	Redo-SVR mitral	Redo-SVR mitral
Size and type of valve replacement	Mechanical valve No. 21	Mechanical valve No. 31	Mechanical valve No. 25
CEC time (min)	152	143	181
Clamp time (min)	81	98	74
Number of previous valvular surgery	1	2	1

Any potential benefit of pre-operative physiologic improvement must be weighed against the risk of known ECMO complications, which are not insignificant. According to a meta-analysis by Cheng *et al*.,^8^ 30.4% of patients receiving VA-ECMO due to CS had a significant infection. VAP was the most common cause, with a variable frequency ranging from 33%^9^ to 55%.^10^ In all three of our cases, infection occurred. In the second case, it was a recurrence of a previously diagnosed endocarditis. In the first and third cases, it was sepsis of pulmonary origin caused by VAP. In both cases, the isolation showed *K. pneumoniae*, and in one, there was a coinfection with *Escherichia coli*, with a frequency of 26% and 9%, respectively, in the literature.^10^ Studies have shown that proper infection control measures, including hand hygiene and routine surveillance cultures, can significantly reduce the incidence of infectious complications in ECMO patients.^11^

Vascular complications are also common. The first case was a morbidly obese patient, and due to the abundant subcutaneous tissue, there was a long path between the skin incision and the vessel. In these cases, the use of rigid wires for cannulation support and the use of accessory vascular accesses such as subclavian or axillary are described. Nevertheless, these are associated with a higher risk of neurological complications.^12^ Despite the use of a rigid wire during cannulation, a false path was created, which resulted in a vascular injury that led to massive bleeding. Therefore, in obese patients, open cannulation may avoid vascular complications.

Regarding surgical wound closure in ECMO patients, the tendency towards hypercoagulability and thrombosis must be considered. Anticoagulation is necessary, seeking a balance between hypercoagulability and bleeding. Early closure can lead to complications such as bleeding and the need for re-exploration, in addition to ECMO-related complications. Therefore, all of our patients had delayed sternal closure (DSC). Our strategy of DSC is based on the use of a VAC KCI® system, where white foam and granufoam are used to protect the great vessels and the soft tissue, respectively. The VAC system is programmed to ensure a continuous negative pressure of 75 mmHg in order to achieve bleeding control by three-dimensional wound compression and promote wound healing.^13^ Delayed sternal closure following prolonged surgery with the use of mediastinal packing and the release of mediastinal adhesions in the case of re-operations aims to prevent or control post-operative bleeding in an oedematous and functionally impaired heart.^14^ It has been proved that it is particularly useful in emergency surgery or in patients with pre-operative bleeding tendencies.^13^ Christenson *et al*.^15^ showed that DSC was effective for patients with cardiac dysfunction, persistent bleeding, and arrhythmias after cardiac surgery without a significant increase in morbidity in a cohort with a rate of 78.9% survival.

The research supporting the use of ECMO to bridge inoperable patients to definitive cardiac procedures is still relatively scarce. That is why decisions in these cases are made on a case-by-case basis. The lack of a control group and the small sample size limit the ability to draw definitive conclusions regarding treatment efficacy. Nevertheless, this descriptive information can serve as a starting point for new hypotheses and upcoming research.

## Conclusion

This case highlights the potential role of ECMO in patients with a redo cardiac operation to optimize haemodynamic status. Although ECMO therapy carries complications such as bleeding and infection with a high mortality rate, it might serve as an intermediate step in recovering patients from shock and giving them a chance of a redo cardiac surgery. Despite patients having high-risk scores on initial presentation for surgery, VA-ECMO has the potential to reduce operative mortality if surgery is performed after haemodynamic stabilization. For obese patients, the femoral cut-down cannulation may prevent false routes and major bleeding.

## Supplementary Material

ytad569_Supplementary_Data

## Data Availability

Data supporting this study are included within the article and/or supporting materials.

## Lead author biography

Dr Alvaro Diego Peña is a consultant adult cardiac surgeon at Fundación Valle del Lili. He is the director of minimally invasive cardiac surgery program.
