# Supplementary figures and images for: Veno-arterial extracorporeal membrane oxygenation as a perioperative support to redo cardiac surgery for inoperable adult patients: a case series
Source: Eur Heart J Case Rep. 2023 Nov 16;7(12):ytad569. doi: 10.1093/ehjcr/ytad569 (PMC10733174; doi:10.1093/ehjcr/ytad569)

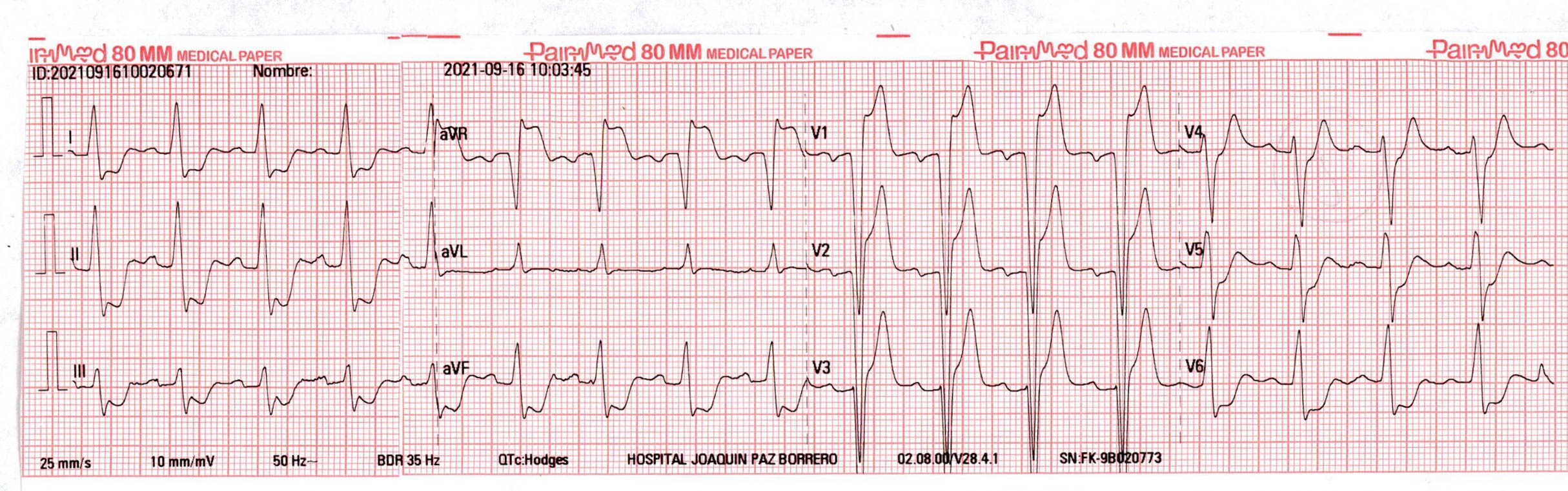

Supplement: ytad569_Supplementary_Data [file ytad569_supplementary_data.jpeg]
